# Associations Between Education and Cognitive Decline Revisited: Evidence From India

**DOI:** 10.1093/geroni/igaf122.1512

**Published:** 2025-12-31

**Authors:** Emma Nichols, Alden Gross, Richard Jones, Erik Meijer, Lindsay Kobayashi, Jinkook Lee

**Affiliations:** University of Southern California, Los Angeles, California, United States; Johns Hopkins Bloomberg School of Public Health, Baltimore, Maryland, United States; Brown University, Brown University, Rhode Island, United States; University of Southern California, Los Angeles, California, United States; University of Michigan, Ann Arbor, Michigan, United States; University of Southern California, Los Angeles, California, United States

## Abstract

Educational attainment is consistently associated with cognitive level, but the evidence for cognitive decline is mixed, with most research suggesting no association. However, most evidence comes from high-income contexts. We used nationally representative data from the first two waves of the Longitudinal Aging Study in India – Diagnostic Assessment of Dementia (LASI-DAD) (N = 3,673) to estimate the association between educational attainment and cognitive decline using linear mixed effects models. We evaluated the consistency of findings across a range of generalized estimating equations (GEE) models with inverse probability weights to adjust for sample selection, attrition, and mortality. We further explored the role of practice effects, selective survival, and regression to the mean using a range of supplementary analyses, including simulation analysis. Compared to those with no education, those with less than primary school (difference of -0.03 SD units/year; 95% CI -0.04 to -0.01), primary school (-0.04; -0.06 to -0.03), middle-secondary school (-0.06; -0.07 to -0.04), and higher secondary school education and up (-0.05; -0.07 to -0.03) had steeper cognitive decline after adjustment for demographic and early-life socioeconomic factors. Neither selective survival nor practice effects could explain findings. However, evidence suggested that due to the strong association between education and baseline cognition, educational attainment may act as a proxy for cognitive level and lead to regression to the mean in models adjusting for educational attainment. Regression to the mean may impact findings on the association between education and cognitive decline more broadly, particularly in contexts with larger associations between education and cognitive level.

